# Morphological and molecular analysis of apoptosis in the corpus cavernosum of rats submitted to a chronic alcoholism model ^1^

**DOI:** 10.1590/s0102-865020200030000005

**Published:** 2020-06-03

**Authors:** Rogério José de Azevedo Meirelles, Fermino Sanches Lizarte, Mucio Luiz de Assis Cirino, Paulo Cezar Novais, Isabella Stracieri Gula, Jairo Pinheiro da Silva, Maria de Fátima Galli Sorita Tazzima, Valéria Paula Sassoli Fazan, Marina Toledo Durand, Daniela Pretti da Cunha Tirapelli, Camila Albuquerque Melo de Carvalho, Bruno César Schimming, Carlos Augusto Fernandes Molina, Silvio Tucci, Luis Fernando Tirapelli

**Affiliations:** I PhD, Department of Surgery and Anatomy, Faculdade de Medicina de Ribeirão Preto, Universidade de São Paulo (FMRP-USP), Ribeirao Preto-SP, Brazil. Acquisition of data.; II PhD, Department of Surgery and Anatomy, FMRP-USP, Ribeirao Preto-SP, Brazil. Technical procedures.; III Fellow PhD degree, Postgraduate Program in Clinical Surgical, Department of Surgery and Anatomy, Surgical Clinic Program, FMRP-USP, Ribeirao Preto-SP, Brazil. Acquisition of data.; IV PhD, Department of Surgery and Anatomy, FMRP-USP, Ribeirao Preto-SP, and Postgarduate Program in Structural and Functional Interactions in Rehabiliattion, Universidade de Marília (UNIMAR), Marilia-SP, Brazil. Histopathological examinations.; Postgarduate Program in Structural and Functional Interactions in Rehabiliattion, Universidade de Marília, Marilia, SP, Brazil; V Graduate student, School of Medicine, Universidade de Ribeirão Preto (UNAERP), Brazil. Acquisition of data.; VI PhD, Department of Surgery and Anatomy, FMRP-USP, Ribeirao Preto-SP, and Professor, Faculdade de Taquaritinga (FTGA), Brazil. Manuscript preparation.; Faculdade de Taquaritinga, Brazil; VII PhD, Assistant Professor, Department of Surgery and Anatomy, FMRP-USP, Ribeirao Preto-SP, Brazil. Manuscript writing.; VIII PhD, Associate Professor, Department of Surgery and Anatomy, FMRP-USP, Ribeirao Preto-SP, Brazil. Manuscript writing.; IX PhD, UNAERP, Ribeirao Preto-SP, Brazil. Manuscript writing.; X PhD, Associate Professor, Department of Surgery and Anatomy, FMRP-USP, Ribeirao Preto-SP, Brazil. Final approval.; XI PhD, Department of Surgery and Anatomy, FMRP-USP, and Professor, Centro Universitário Barão de Mauá, Ribeirao Preto-SP, Brazil. Manuscript writing.; Centro Universitário Barão de Mauá, Ribeirao Preto, SP, Brazil; XII PhD, Department of Surgery and Anatomy, FMRP-USP, Ribeirao Preto-SP, and Associate Professor, Department of Bioscience, Universidade Estadual Paulista (UNESP), Botucatu-SP, Brazil. Critical revision.; Department of Bioscience, Universidade Estadual Paulista, Botucatu, SP, Brazil

**Keywords:** Ethanol, Erectile Dysfunction, Apoptosis, MicroRNAs, Rats

## Abstract

**Purpose:**

To evaluate the effect of chronic alcoholism on morphometry and apoptosis mechanism and correlate with miRNA-21 expression in the corpus cavernosum of rats.

**Methods:**

Twenty-four rats were divided into two experimental groups: Control (C) and Alcoholic group (A). After two weeks of an adaptive phase, rats from group A received only ethanol solution (20%) during 7 weeks. The morphometric and caspase-3 immunohistochemistry analysis were performed in the corpus cavernosum. The miRNA-21 expression was analyzed in blood and cavernous tissue.

**Results:**

Chronic ethanol consumption decreased cavernosal smooth muscle area of alcoholic rats. The protein expression of caspase 3 in the corpus cavernosum was higher in A compared to the C group. There was no difference in the expression of miRNA-21 in serum and cavernous tissue between the groups.

**Conclusion:**

Chronic ethanol consumption reduced smooth muscle area and increased caspase 3 in the corpus cavernosum of rats, without altered serum and cavernosal miR-21 gene expression.

## Introduction

Penile erection is a set of physiological events, including psychical, neural and vascular mechanisms, which involves relaxation of vascular smooth muscle by means of neural stimulation and an increase in arterial blood flow of the corpus cavernosum^1^ . When one of these events does not happen or is insufficient, it promotes an inability to achieve or maintain the proper penile erection for sexual satisfaction called erectile dysfunction (ED)^2^_._

Chronic alcoholism represents an important risk factor for ED since high levels of alcohol decrease penile tumescence and reduce sexual performance^3^ . Ethanol-induced sexual dysfunction is caused by oxidative stress in the endothelium and vascular smooth muscle cells and its effect on the autonomic nervous system^4^ . The association of excessive ethanol consumption and apoptosis process is also frequently explored in literature. A study demonstrated that excessive ethanol consumption caused a marked thinning of the left ventricular wall associated with high caspase3 activity in rats, indicating high level of apoptosis^4^ .Studies reported the occurrence of histopathological alterations and increase of the caspase 3 and 9 proteins expression in the cerebellar cortex of rats submitted to the chronic alcohol model^5 , 6^ .

The mechanism of apoptosis with increased expression of caspase 3 has also been described in corpus cavernosum of diabetic rats and aged rats^7 , 8^ . Hence, it is believed that there is a relationship between ED and alcohol consumption^9^ . In fact, reports of the occurrence of the mechanism of apoptosis in the corpus cavernosum are associated with denervation, castration, alcoholism and chronic diseases such as diabetes^10 , 11^ .

A large number of biological processes are modulated by microRNAs (miRNAs), small molecules of RNA that regulate post-transcriptional silencing of target genes^12^ . Among them, miR-21 was considered one of the top 20 differentially expressed microRNAs screened using the Morpheus online tool^13^ . Several studies have shown that miR-21 regulates a series of biological events in heart disease and cancer, including cell proliferation, migration, invasion, metastasis, apoptosis and fibrosis^14 , 15^ .

In preclinical studies, exogenous miRNA-21 sent into cardiomyocytes and endothelial cells in myocardial infracted mice, drastically inhibited cell apoptosis and improved cardiac function^16^ .In cancer patients, some studies show that miR-21 is closely associated with the evolution of prognosis, recurrence and diagnosis^17^ .Since MiR-21 has a strong antiapoptotic action and therefore inhibits cell death, we hypothesize that chronic alcoholism could affect miRNA-21 which contribute to apoptosis mechanism in the corpus cavernosum of rats. Therefore, the objective of this study was to evaluate the effect of chronic alcoholism on morphometry and apoptosis mechanism and correlate with miRNA-21 expression in the corpus cavernosum of rats submitted to a model of “semi-voluntary alcoholism”.

## Methods

A total of 24 male Wistar rats ( *Rattus norvegicus* ) from the Universidade de São Paulo, Ribeirão Preto Campus, after approval by the Ethics Committee of our institution, were used. They were divided in 2 groups and followed by 4 weeks after the adaptive period: control group (C) and alcoholic group (A), all groups consisting of 12 animals each.

The model of “semi-voluntary alcoholism” proposed by Tirapelli *et al* .^18^ was used. After two weeks of an adaptive period, increasing weekly the concentrations of ethanol (5, 10, 20%), the experimental phase started in the third week of treatment. The rats received only ethanol solution at 20% during 7 weeks and then were euthanized.

### Morphometric and immunohistochemistry analysis

For the morphometric and immunohistochemistry analysis, the corpus cavernosum of control (n = 6) and alcoholic (n=6) were immediately removed and fixed for 24 h in ice-cold 0.1 mol/l PBS (pH 7.4), containing 4% paraformaldehyde, followed by cryoprotection in 15% of sucrose for 4 h and 30% sucrose overnight at 4°C. The samples (longitudinal sections (3 µm) of the corpus cavernosum) were embedded in paraffin and stained with Masson’s Trichrome Technique. The following morphometric analyses were performed in the corpus cavernosum of six animals from each experimental group (C and A):

- Footprint (in μm^2^) by the smooth muscles of the corpus cavernosum. This analysis was performed throughout the length of the corpus cavernosum.
- Footprint (in μm^2^) by gaps or cavernous spaces. This analysis was performed for five fields chosen from the anterior and posterior regions of each corpus cavernosum and in the central portion there between.
- Footprint (in μm^2^) by the collagen fibers of the connective tissue of the corpus cavernosum. This analysis was performed from the five fields chosen for the previous assessment (area of the cavernous spaces).

Thus, from the same fields chosen for the evaluations B and C, the value of the total area occupied by each field in x400 magnification (the default value of 94019.38 μm^2^) was originally obtained, and then the total amount occupied by the cavernous spaces was captured. Thus, the pattern occupied by the field value was subtracted from the amount occupied by the cavernous spaces, which is the equivalent to the area of value mainly occupied by collagen fibers, but also by elastic fibers and smooth muscle cells (total area of these three histological constituents).

Longitudinal sections (3 µm) of the corpus cavernosum were immunohistochemically analyzed via avidin-biotin-peroxidase (Novostain Super ABC Kit - Universal, NCL-ABCu, Novocastra Laboratories Ltd, Newcastle upon Tyne, UK) - (universal Kit mach 4 BIOCARE). The longitudinal sections were incubated with 3% H_2_O_2_, followed by antigen retrieval with a moist heat steam cooker Optistream Plus (Krups North America, Inc., Millville, New Jersey, USA) with 10 mM citrate buffer at pH 6.0 for 35 minutes. Then, the sections were incubated for 24 hours in a primary antibody: Caspase-3 diluted 1/300 in PBS solution of bovine serum albumin (BSA). Subsequently, the blocking of the endogenous biotin was performed (Biotin Blocking System, Dako North America, Inc., Carpinteria, USA) and only then the sections were incubated with secondary antibody HRP kit MACH 4-Universal Polymer (M4BD534, Biocare Medical) and then with avidin-biotin-peroxidase kit same (1/200 in PBS). Color was developed by the addition of diaminobenzidine (Sigma Chemical, St. Louis, USA).

The sections were dehydrated in ethanol, cleared with xylene and mounted under the cover slip with Permount liquid (Fisher Scientific Company LLC, Fair Lawn, New Jersey, USA).

To evaluate the background reaction, the procedures were also performed in sections incubated only with the secondary antibodies (indirect technique) or in the absence of antibodies (direct technique).

The slides for morphometric and immunohistochemistry were analyzed using the Zeiss microscope Axioskop 2 plus model in magnification of 400 times. The number of cells with positive staining for Caspase-3 was measured by using a camera (Axio Cam, Zeiss, Germany) and the program Axiovision 4.6 (Zeiss, Germany).

### Analysis of expression profile of the miRNA-21

The expression profile of the miRNA-21 was analyzed in blood and the cavernous tissue samples from each animal. Total cellular RNA was extracted using Trizol Reagent (Invitrogen, Carlsbad, CA) and RNA was reverse transcribed to single-stranded cDNA, using a High Capacity Kit (Applied Biosystems, Foster City, CA) according to the manufacturer’s protocol. For quantitative analysis of the miRNA-21 (002493), we used the commercially available system TaqMan Assay-ondemand (Applied Biosystems). Reverse transcription was performed using 5ng total RNA for each sample in 7,5µL of the total reaction mixture. The cDNA obtained was diluted 1:4 and 4.5µL was used for each 10µL of the quantitative real-time polymerase chain reaction mixture using the TaqMan Master Mix (Applied Biosystems). All reactions were carried out in duplicate and analyzed with the 7500 Sequence Detection System apparatus (Applied Biosystems). Data were analyzed using the ABI-7500 SDS software. The total RNA absorbed was normalized on the basis of the Ct value for U6 (000391). The variation in expression among samples was calculated by the 2-∆∆Ct method, with the mean ∆Ct value for a group of 6 samples from control rats being used as a calibrator.

### Statistical analysis

For the evaluation of all studies of this research (morphometric, protein expression and gene expression), statistical analysis was performed using Two-way ANOVA and Dunns Pos Hoc or T Test and Mann Whitney post-test. We used the GraphPad Prism program 6:00 version for Windows (GraphPad Software, San Diego - California USA) and considered statistically significant p values <0,05.

## Results

**Figure 1:**
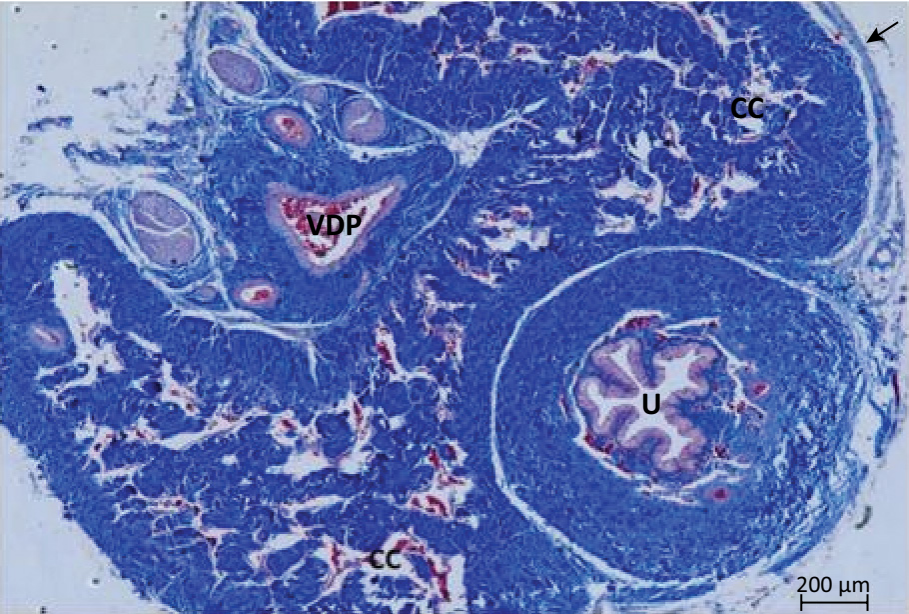
Cross-sectional photomicrograph of the rat penis where the two corpus cavernosum (CC) covered by the tunica albuginea ( *arrow* ) can be identified and contain a large number of cavernous ( *blank* ) spaces within it; ventrally the urethra (U) inside the spongy body and the dorsal deep vein of the penis (VDP). Masson’s trichrome, x50.

**Figure 2:**
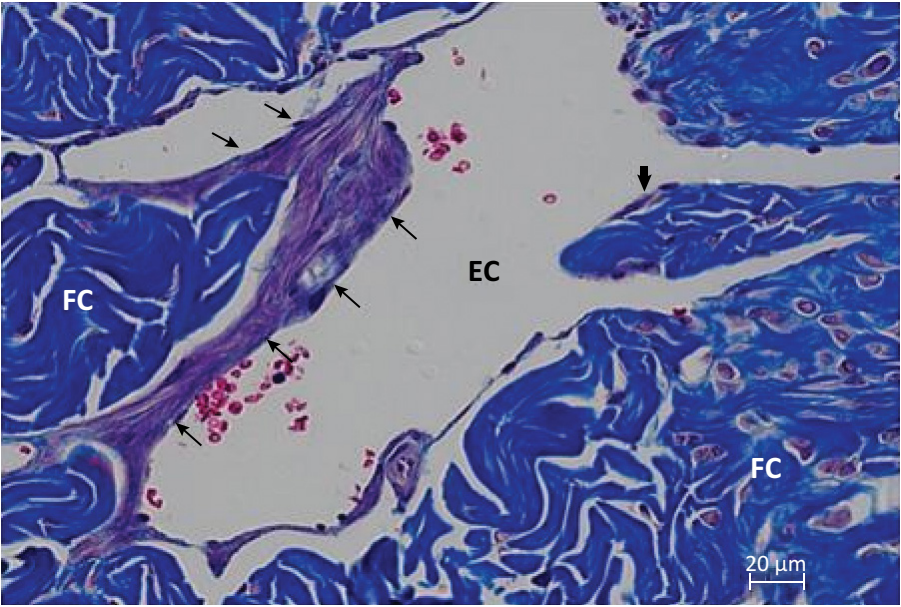
Photomicrography showing detail of some connective trabeculae composed mainly of collagen fibers (CF) in blue and by subendothelial smooth muscle ( *thinner arrows* ). In the center a large cavernous space (EC) containing red blood cells inside ( *red* ) and endothelial ( *thick arrow* ). Masson’s trichrome, x400.

In the region of the middle segment of the cavernous body of the albino *Wistar* rat penis, from a cross section, we could identify two cavernous cylinders with extensive communication with each other. It is noted that the cavernous body is composed of a strong conjunctive membrane, the tunica albuginea, from which traves delimit gaps of varying size and shape ( Fig. 1 ). The root of the corpus cavernosum presents trabeculae consisting predominantly of smooth muscle tissue, endothelium lined with large gaps of varying shapes and sizes. Some collagen fibers are also observed; however, in a larger quantity in the trabeculae of the region of the body. In this portion, smooth muscle fibers are found sparsely. The gaps are smaller when compared to the root region and also delimited by endothelium ( Fig. 2 ).

**Figure 3:**
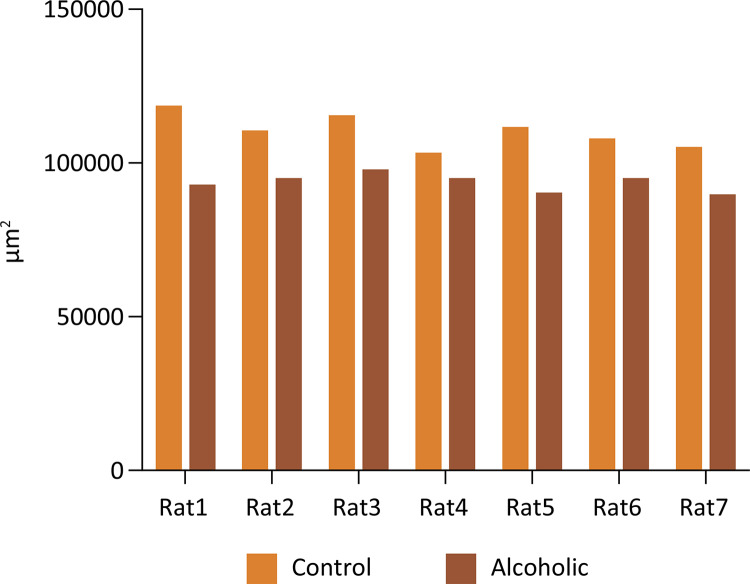
Average area occupied by the smooth muscle fibers of the corpus cavernous of each animal from A and C groups (Two- way ANOVA, p=0.0002).

**Figure 4:**
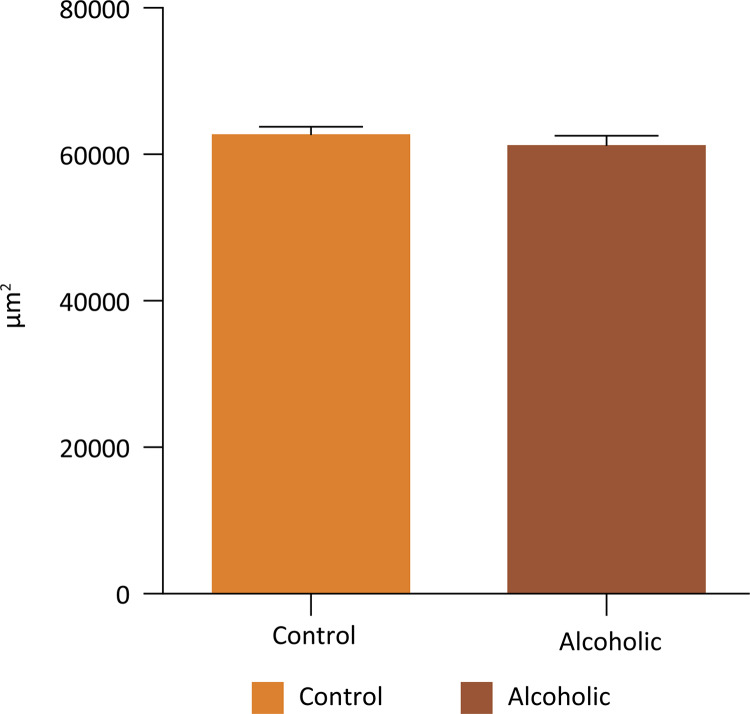
Average area occupied by collagen fibers corpus cavernosum of the C and A groups (Mann-Whitney test, p=0.1988).

**Figure 5:**
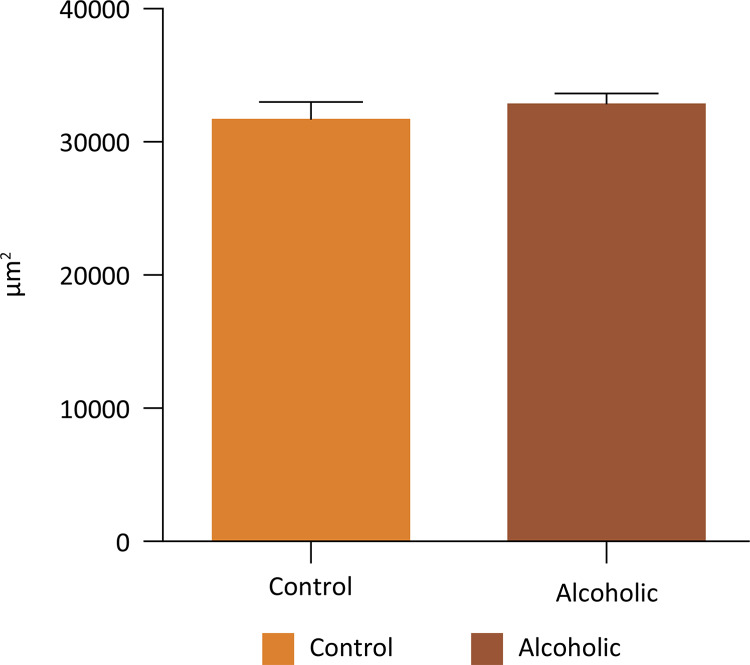
Average area occupied by gaps or cavernous spaces of the corpus cavernosum in groups C and A (Mann-Whitney test, p=0.1583).

In the morphometric evaluation of the smooth muscle area of the corpus cavernosum (in μm^2^), we observed a significant decrease in A group compared to the control group (p = 0.0002, two- way ANOVA) ( Fig. 3 ). There was no difference between the occupied area (in μm2) by collagen fibers (Mann- Whitney test, p = 0.1988) and cavernous spaces (Mann- Whitney test, p = 0.1583) of C and A groups ( Figs. 4 and 5 ).

**Figure 6:**
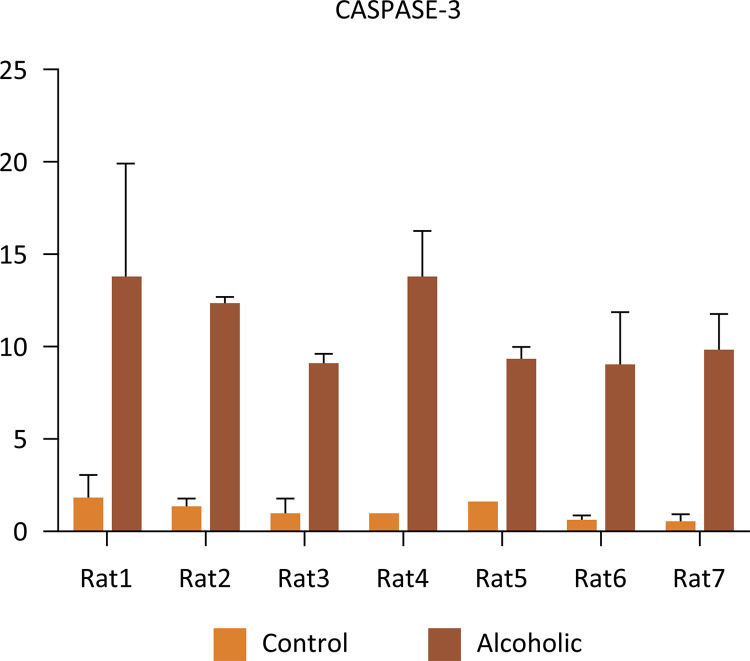
Average of the protein expression of caspase 3 in the corpus cavernosum of C and A groups (Two-way ANOVA, p<0.0001).

**Figure 7:**
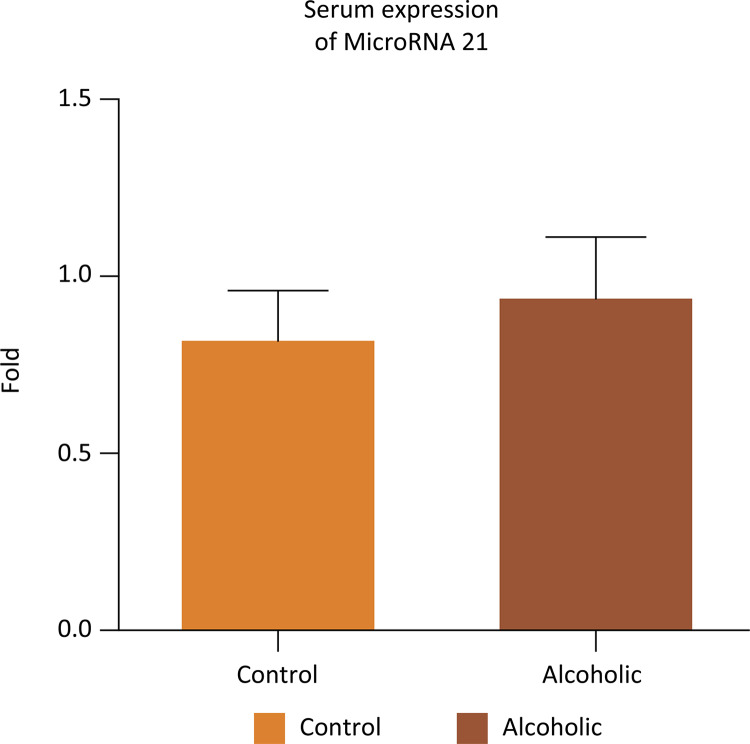
Gene expression of miRNA -21 in the serum in groups C and A (Mann- Whitney test, p=0.2135).

**Figure 8:**
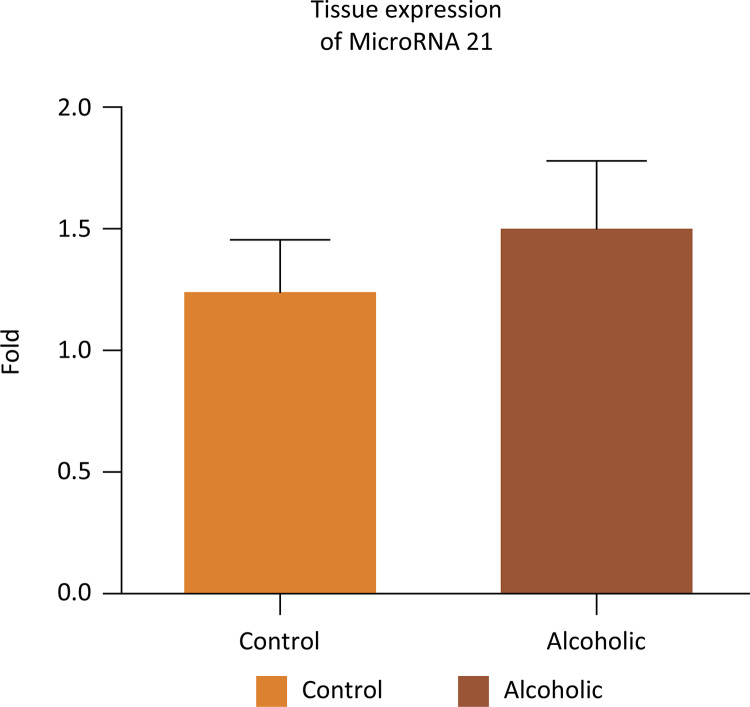
Gene expression of miRNA -21 in the corpus cavernosum tissue in groups C and A (Mann- Whitney test, p=0.864).

The protein expression of caspase 3 in the corpus cavernosum was higher in A when compared to the C group ( Fig. 6 ) (two- way ANOVA, p < 0.0001). The serum and cavernous tissue expression of the miR-21 were shown in Figures 7 and 8 . We observed lower expression of the miR-21 in both groups without significant difference between them (Mann-Whitney test, serum: p = 0.2135, tissue: p = 0.864).

## Discussion

Chronic alcoholism is a known risk factor for sexual dysfunction and has been well described for a long time^3^ . Accordingly, our data demonstrated that rats submitted to a chronic alcohol model presented significant decrease in smooth muscle area of the corpus cavernosum associated with increase in caspase 3 and no alteration in miR-21 gene expression.

Smooth muscle fibers contribute to the rigidity and stability of the erect penis to increase intracavernous pressure, which could not be achieved only by a vascular mechanism^19^ . An experimental study found that ethanol significantly affects the contraction and relaxation of the smooth muscle of the cavernous body of rabbits^20^ . In men with ED, there is evidence that the content of smooth muscle cells and elastic fibers in cavernosal tissue is reduced compared to men without ED^21^ .

In rats, fewer numbers of smooth muscle cells and collagen and elastic fibers in the corpus cavernosum of alcoholic animals were also observed when compared to control^22^ . Furthermore, it was observed that mice treated with ethanol during 14 days showed deep cuts in the nuclear membrane of endothelial cells, reduction of vesicles of pinocytosis and vacuolization in the cytoplasm of some endothelial cells of the corpus cavernosum. Thus, these data together with ours indicate that alcoholism affects the integrity of smooth muscle and endothelial cells of the corpus cavernosum, which can be one of the mechanisms responsible for ED^23^ .

Previous studies from our group have also described the consequences of alcohol use on ED showing that diabetic and alcoholic rats presented blunted nitric oxide-mediated relaxation associated with increased contractile sensitivity to noradrenergic nerve stimulation in the cavernosal smooth muscle and decreased serum testosterone^25 , 26^ . Recently, we demonstrated that chronic ethanol consumption associated with diabetes played a role in the pathogenesis of ED by means of the reduction in the turnover of ETa and ETb receptors of endothelin-1, a potent vasoconstrictor peptide, and in the miRNA-155 and miRNA-199 levels in corpus cavernosum of rats^27^ .

Several studies also associate the expression of different miRNAs with ED. A recent study identified that the expression of four miRNAs, miR-1, miR-200a, miR-203 and miR-206, were significantly upregulated in the corpus cavernosum of rats with ED. It is speculated that these four positive expression miRNAs play a crucial role in the regulation of the eNOS / NO / PKG and PGE1 / PKA pathways and are subsequently involved in the development of aging-related ED^28^ . In our study, we did not observed alteration in miRNA-21 expression in the corpus cavernosum and serum of alcoholic animals even with an increase in caspase 3. It is likely that miRNA-21 does not play a role in the ED induced by chronic alcoholism.

Some important mechanisms suggested as responsible for ED in diabetes involve increased apoptosis, collagen deposition and reduced smooth muscle content in the corpus cavernosum. Studies have shown that impaired erectile function in diabetic rats is associated with over-activation of penile PARP pathway, upregulation of apoptosis-related proteins, including Bax and caspase-3, and downregulation of anti-apoptotic factors^29^ .

Caspases are proteases with a well-defined role in apoptosis^30^ . Studies have demonstrated that caspase-3 is activated in several models of ED e.g., aging, cavernous nerve injury, hyperhomocysteinemia, radiotherapy^31^ . Our data also demonstrated an increased caspase-3 expression in the corpus cavernosum of rats submitted to the chronic alcohol model. This indicated that caspase-3 is involved in the reduction of smooth muscle area in ethanol-induced sexual dysfunction.

## Conclusions

Chronic alcoholism decreased cavernosal smooth muscle area and increased caspase 3 in rats, indicating a higher apoptosis process in this animal model of ED. Nevertheless, miR-21 gene expression in serum and corpus cavernosum was not affected by ethanol consumption.

## Footnotes

1 Research performed at Molecular Biology Laboratory, Department of Surgery and Anatomy, Medical School of Ribeirao Preto, Universidade de São Paulo (USP), Brazil.
